# Multiunit Activity-Based Real-Time Limb-State Estimation from Dorsal Root Ganglion Recordings

**DOI:** 10.1038/srep44197

**Published:** 2017-03-09

**Authors:** Sungmin Han, Jun-Uk Chu, Hyungmin Kim, Jong Woong Park, Inchan Youn

**Affiliations:** 1 Biomedical Research Institute, Korea Institute of Science and Technology, 5, Hwarang-ro 14-gil, Seongbuk-gu, Seoul, 02791, Korea; 2 Department of Biomedical Science, Korea University College of Medicine, 73, Inchan-ro, Seongbuk-gu, Seoul, 02841, Korea; 3 Daegu Research Center for Medical Devices and Rehabilitation Engineering, Korea Institute of Machinery and Materials, 330, Techno Sunhwan-ro, Yuga-myeon, Dalseong-gun, Daegu, 42994, Korea; 4 Department of Biomedical Engineering, Korea University of Science and Technology, 217, Gajeong-ro, Yuseong-gu, Daejeon, 34113, Korea

## Abstract

Proprioceptive afferent activities could be useful for providing sensory feedback signals for closed-loop control during functional electrical stimulation (FES). However, most previous studies have used the single-unit activity of individual neurons to extract sensory information from proprioceptive afferents. This study proposes a new decoding method to estimate ankle and knee joint angles using multiunit activity data. Proprioceptive afferent signals were recorded from a dorsal root ganglion with a single-shank microelectrode during passive movements of the ankle and knee joints, and joint angles were measured as kinematic data. The mean absolute value (MAV) was extracted from the multiunit activity data, and a dynamically driven recurrent neural network (DDRNN) was used to estimate ankle and knee joint angles. The multiunit activity-based MAV feature was sufficiently informative to estimate limb states, and the DDRNN showed a better decoding performance than conventional linear estimators. In addition, processing time delay satisfied real-time constraints. These results demonstrated that the proposed method could be applicable for providing real-time sensory feedback signals in closed-loop FES systems.

Functional electrical stimulation (FES) has been investigated to restore lost functions in paralyzed patients suffering from stroke, spinal cord injury, and multiple sclerosis^1^. In FES systems, paralyzed muscles are modulated by electrical stimulation that can activate peripheral nerves and generate contractions^2^. However, electrical stimulation frequently causes muscle fatigue and tissue damage^3^,^4^. Additionally, most FES systems have a limitation in that they require continuous or repeated user input to provide suitable feedback signals. Natural motor functions are controlled by the sensory-to-motor transformation, where a motor command is generated based on sensory feedback, such as that from tactile and proprioceptive afferents^5^. Thus, appropriate feedback signals are required for achieving complex and natural control in FES systems, and many studies have been performed to extract reliable and relevant sensory information from artificial or natural sensors^6^,^7^,^8^.

A neural interface is a useful technology for extracting sensory information from the nervous system and providing feedback signals for use in FES systems. The sensory signals generated by natural sensors represent detailed and intuitive sensory information, including body movements, posture, and behaviour, and these neural activities can be recorded from the peripheral or central nervous system using a microelectrode. Dorsal root ganglia are attractive locations for obtaining natural sensory information because dorsal root ganglion neurons transmit somatosensory information about a variety of modalities, such as pressure, vibration, and pain. Many studies have investigated how to decode sensory feedback from the afferent signals recorded from dorsal root ganglia^9^,^10^,^11^,^12^,^13^,^14^. These studies utilized simultaneous extracellular recordings from up to 100 electrodes, and achieved a high level of decoding accuracy.

When using a multichannel microelectrode to record neural activities, spike sorting must be performed to discriminate the signals from individual neurons (spikes) from the overall signal from multiple neurons. However, current spike sorting algorithms are limited in their processing efficiency and reliability in cases involving overlapping spikes, bursting neurons, and electrode drift. In addition, the computational cost required to improve spike sorting performance is high^15^,^16^. In evaluating the complementary aspects of decoding accuracy and computational complexity, several studies have suggested an alternate decoding paradigm strategy using multiunit activity instead of single-unit activity^17^,^18^,^19^. For example, multiunit activity recorded from the primary motor cortex has been used to extract information about movement intent without using conventional spike sorting or carefully setting threshold values, and unsorted threshold-crossing values were calculated for all events crossing a negative threshold^17^. Another study predited hand movements in macaques using multiunit activity, which was calculated based on the root mean square for the frequency band between 200 and 6000 Hz for neural activity recorded from the primary motor or premotor cortex^19^. These studies used superimposed neural activities to extract control signals from intracortical recordings and achieved a better decoding performance than methods using traditional spike sorting approaches. Such multiunit activity-based decoding approaches are also applicable for extracting sensory information from afferent signals to provide feedback signals for use in closed-loop FES systems. We have previously demonstrated continuous, real-time sensory event detection from tactile afferent signals using a combination of various multiunit activity features and a multilayer perceptron classifier^20^. However, in this previous study, we assumed that relatively simple sensory events were generated by the mechanical stimulation of three different areas of the rat hind paw. The relationship between tactile afferent signals and sensory events was constructed using a static linear model; therefore, detection performance was guaranteed only for discrete sensory events. Addtionally, this approach has limitations in its application to practical FES systems as it does not consider interference from stimulus artefacts that result from electrical stimulation and comtaminate neural signals. During natural motor functions, such as standing or walking, recorded afferent signals include tactile and proprioceptive information. Therefore, to estimate limb states, a decoding method should extract sensory information from proprioceptive afferent signals and be able to describe a dynamic relationship between proprioceptive afferent signals and different limb states. Additionally, a method to eliminate stimulus artefacts is necessary to obtain interference-free neural signals.

Addressing these issues, the current study presents a multiunit activity-based decoding method for providing real-time limb-state feedback to allow for closed-loop control in FES systems. When neural signals are recorded during electrical stimulation, stimulus artefacts appear in the recording channels and make it difficult to analyse neural responses. Although stimulus artefacts can be effectively eliminated via blanking for a period of time during stimulation, this results in the loss of information and decreases the available signal length. Accordingly, a robust and accurate feature extraction method is necessary for extracting sensory information from artefact-rejected neural signals. Feature vectors can be extracted based on mean absolute values (MAVs) which are easy to compute and able to accurately represent the activity levels of residual signals in subsequent blanking. A decoding method should be able to represent nonlinear dynamic relationships between proprioceptive afferent signals and limb states. To estimate limb states, we used a dynamically driven recurrent neural network (DDRNN). This DDRNN involves dynamic observer-based output feedback that is fed to the neural network with a time delay. As a result, the feedback loop creates internal dynamics, enhancing the decoding performance.

In this study, multiunit activity-based feature vectors were extracted from proprioceptive afferent signals, which were recorded using a multichannel microelectrode at the dorsal root ganglion of the seventh lumbar vertebra (L7) during passive movements of the ankle and knee joints. Ankle and knee joint angles were then estimated by the DDRNN from the MAVs of multiunit activity signals. The decoding accuracy of the proposed method was compared with the accuracy of other feature extraction methods and estimators. The multiunit activity-based feature vector, MAV, exhibited an accuracy similar to that of a single-unit activity-based feature vector, and the proposed nonlinear recursive estimator, DDRNN, was significnatly more accurate than linear estimators. Additionally, the processing time delay of the proposed method was enough to meet real-time constraints. These results suggest that the proposed multiunit activity-based decoding method is a useful approach to providing limb-state feedback for closed-loop control in FES systems.

## Results

### Properties of Recorded Proprioceptive Afferent Signals

**Figure 1:**
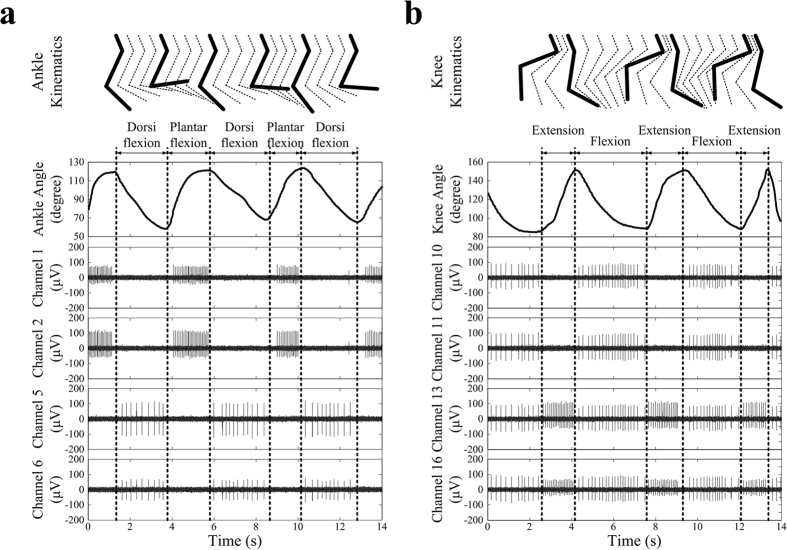
Typical examples of recorded afferent signals from the right seventh dorsal root ganglion. (**a**) Recorded proprioceptive afferent signals during ankle joint movement alone. (**b**) Recorded proprioceptive afferents signals during knee joint movement alone. Different signal patterns were generated in different electrode channels by ankle and knee joint movements.

Figure 1 shows representative examples of recorded neural signals from the L7 dorsal root ganglion during passive movements of the ankle and knee joints in rabbit A. For ankle joint movements, neural activities were observed at different channels; channels 1 and 2 were activated during ankle dorsiflexion, whereas channels 5 and 6 were activated during ankle plantarflexion. For knee joint movements, channels 10 and 11 were activated during knee flexion, whereas channels 13 and 16 were activated during both knee extension and flexion, and firing activity was increased during extension compared with that during flexion. When a multichannel microelectrode is used to record neural signals from a dorsal root ganglion, the recordings of proprioceptive afferent signals have been found to depend on stochastic variables, such as the positioning of the electrode and the distribution of the sensory neurons^20^. Accordingly, different temporal patterns of neural activities corresponding to different sensory neurons were observed in different electrode channels depending on the joint movements. These signal features could be repeatable and may thereby facilitate the decoding of limb states.

### Comparison of Different Feature Extraction Methods

Table 1 shows the spike sorting results for each of the five rabbits. A 16-channel microelectrode was implanted in the L7 dorsal root ganglion, and the proprioceptive afferent signals were simultaneously recorded from 9 to 14 channels for each electrode. Multiple neurons were recorded on each channel, and 2 to 4 individual neuron signals were isolated per channel. As a result of the spike sorting, the total number of individual neurons obtained for each rabbit was between 36 and 44.

Tables 2 and 3 show the decoding accuracy of the different feature vectors for ankle and knee joint angle estimations, respectively. The decoding accuracy of the MAV feature was compared to those of the MUS and SUS features. For ankle angle estimations, the MAV feature achieved a significantly higher decoding accuracy than the MUS feature (*p* < 0.05), whereas the MAV and SUS features showed similar decoding accuracies (*p* > 0.05). For knee angle estimations, the decoding accuracy of the MAV feature was approximately 0.415% and 0.002% higher than those of MUS and SUS features, respectively, but the differences were not significant (*p* > 0.05). This result suggests that the multiunit activity-based MAV feature vector can perform similarly to or significantly better than the MUS feature vector and, similarly to the conventional spike sorting-based SUS feature vector. This study focused on extracting the most informative sensory information while maintaining a low computational effort to estimate limb states. Thus, the MAV feature was selected as an optimal choice in our decoding application.

### Comparison of Different delay Orders

Tables 4 and 5 show the decoding accuracies of the different delay orders for ankle and knee joint angle estimations, respectively. For ankle angle estimations, the decoding accuracy of the three-order time delay was approximately 8.26% (*p* < 0.05), which was higher than that of the one-order time delay. For knee angle estimations, the three-order time delay also had a higher decoding accuracy, by approximately 4.17%, than the one-order time delay (*p* < 0.05). Meanwhile, there were no significant differences in the decoding accuracies among the three-, five-, and seven-order time delays for both ankle and knee joint angle estimations. The decoding accuracy was not improved by the addition of a delay order of more than three. This result indicates that a three-order time delay for input and output feedback sufficiently improved the decoding accuracy in our experiments.

### Comparison of Different Decoding Methods

Tables 6 and 7 show the decoding accuracy of different decoding methods for estimating ankle and knee joint angles, respectively. The proposed DDRNN was superior to the MLR and Kalman filter. For ankle angle estimations, the decoding accuracy of the DDRNN was, on average, 46.77% (*p* < 0.01) better than the MLR and 39.41% (*p* < 0.01) better than the Kalman filter. For knee angle estimations, the DDRNN also had a higher average decoding accuracy than the MLR (45.62%, *p* < 0.01) and the Kalman filter (39.51%, *p* < 0.01). The MLR could not generalize the nonlinear dynamic relationship of the feature vectors associated with the ankle and knee joint angles. As a linear recursive estimator, the Kalman filter was able to learn the temporal dynamic relationship between the feature vectors and joint angles. However, the Kalman filter predicted the ankle and knee joint angles based on a linear model, leading to low a decoding accuracy.

### Decoding Performance of the Proposed Method

**Figure 2:**
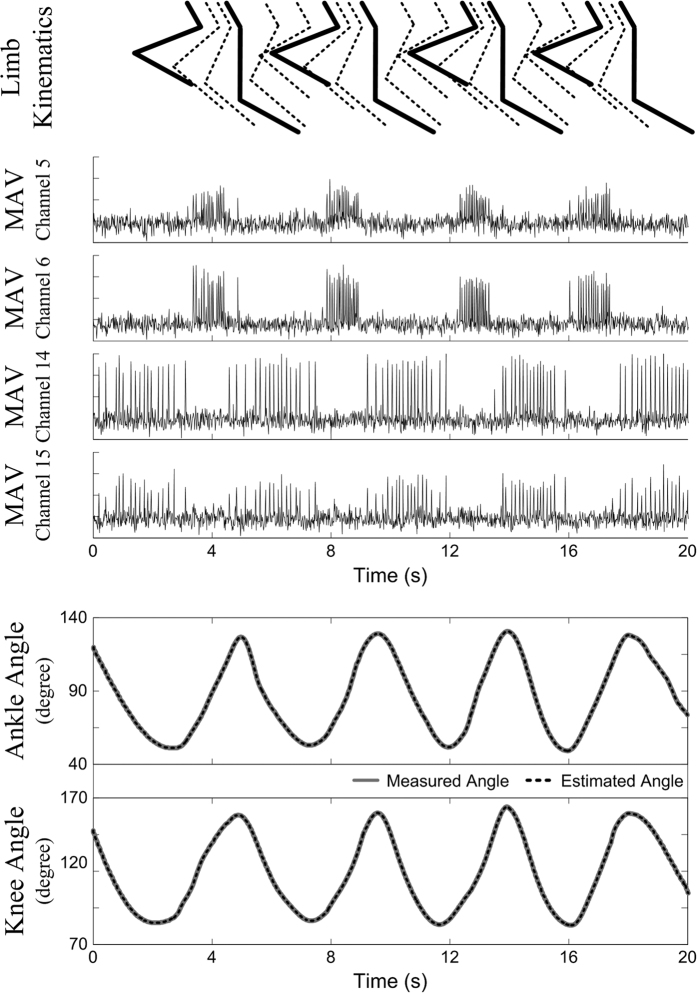
Typical example of decoding results from rabbit E using the proposed method. The top plot is a schematic of the limb kinematics. The middle plot shows the MAV feature vectors of representative channels. The bottom plot shows the ankle and knee joint angle estimates. The grey solid line represents the sensor-measured angles, and the black dashed line represents the estimated angles.

Figure 2 shows an example of a decoding results obtained using the proposed method. The top plot shows the kinematics of the limb movements, while the second plot shows the MAV features of representative channels. Two types of burst activity patterns that depended on ankle and knee joint movements were repeatedly observed: the burst patterns of channels 5 and 6 appeared during ankle dorsiflexion and knee flexion, and the burst patterns of channels 14 and 15 occurred during ankle plantarflexion and knee extension. The activities of sensory neurons corresponding to proprioceptive afferents primarily depend on the kinematic state of one or two adjacent joints^12^. Accordingly, it is predicted that the burst patterns of channels 5 and 6 were proprioceptive afferent activities from the hamstrings and the tibialis anterior, whereas the burst patterns of channels 14 and 15 were proprioceptive afferent activities from the quadriceps and triceps surae. The bottom plot shows the estimation results obtained using the DDRNN for the ankle and knee joint angles. The grey solid line denotes the measured angle data, and the black dashed line denotes the estimated angle data. The *R*^*2*^ values between the measured and estimated angles were 0.99954 and 0.99900 for the ankle and knee joints, respectively.

Table 8 lists the total processing time of the proposed method for estimating ankle and knee joint angles. To implement real-time sensory feedback for closed-loop control in a FES system, the processing time delay should be less than 33.33 ms, which consists of a 15.66-ms data window and a 16.66-ms decision interval. The ankle and knee joint movements were decoded with 30.20 ms. This result indicates that the proposed method can be used for providing limb-state feedback for closed-loop FES systems in real time.

## Discussion

Robust and reliable feedback information regarding the limb state is required for closed-loop control in FES systems. In previous studies, a multi-shank array microelectrode has been used to record the neural signals from one or more dorsal root ganglia, which could allow the recording of hundreds of channels simultaneously^9^,^10^,^11^,^12^,^13^,^14^. This higher number of channels can provide more neural information, and it may potentially lead to improved decoding performance. However, computational effort and task complexity are proportional to the number of channels. Additionally, the used of a multi-shank array microelectrode increases the incidence of deleterious reactive tissue responses, such as foreign-body responses and inflammation^21^. Consequently, one important factor in the neural decoding is obtaining the most informative data from neural signals using the fewest number of shanks and channels^22^. In this study, proprioceptive afferent signals were recorded using a single-shank multichannel microelectrode placed in the L7 dorsal root ganglion, which was selected to record neurons in the femoral and sciatic nerves simultaneously. This approach could reduce computational effort and tissue injury to levels below those associated with multi-shank array microelectrodes.

Spike sorting can be considered a mandatory step to extract information from extracellular recordings because microelectrodes tend to detect the superimposed activity of multiple neurons. The classical approach of spike sorting is performed in several steps. The first step is spike detection, which is usually performed using a threshold method to detect spikes from band-pass filtered signals. The second step is feature extraction, where spike features are extracted based on their shapes. The final step is clustering, which discriminates different spikes by grouping the same spikes in the feature space^15^,^16^. These approaches assume that each neuron produces a reproducible waveform with a different shape. However, current spike-sorting algorithms encounter many challenging problems. Waveforms are easily contaminated by additive noise such as white Gaussian noise and motion artefacts. Additionally, heartbeats and respiration can cause small movements in the location of the electrode, changing waveform shapes. Moreover, electrode viability is important for long-term applications, and signal quality is degraded by biological response, such as tissue growth around the electrode and protein absorption on the electrode, causing spike amplitudes decline and gradually reducing the signal-to-noise ratio (SNR) over time. Spike sorting performance can be unreliable under low SNR conditions, and as a result, high-performance spike sorting is difficult to obtain over a long period. The proposed method achieved a high decoding accuracy using the MAV feature, which was extracted based on multiunit activity data. Multiunit activity data represents neural information that is easy to obtain and is more stable over longer periods of time than single-unit activity data^19^. Therefore, the proposed MAV feature based decoding method could provide more reliable and robust neural information than individual spike activity-based method and could be used to maintain a high level of decoding accuracy during typical long-term recordings.

The proposed DDRNN estimator exhibited a significantly better decoding accuracy than the MLR and Kalman filter estimators. MLR predicts output variables based on multiple input variables, while a Kalman filter predicts a future state based on a linear model of present observations and a prediction of the present state, which has the advantage of being able to effectively learn the temporal dynamics of a system. However, MLRs and Kalman filters model the relationship between input and output variables via a linear equation, making it difficult to estimate nonlinear dynamic relationships. The proposed DDRNN was constructed with a time delay for input and for output feedback, which can be used to assess a nonlinear, dynamic relationship between proprioceptive afferent signals and ankle and knee joint angles and can improve decoding performance.

For closed-loop control in FES applications, corrective feedback signals should be decided upon based on sensory information in real-time. In this study, the scheme for artefact rejection and data-window segmentation were proposed assuming the generation of limb movement via electrical stimulation. The stimulus artefact was eliminated using a blanking processes synchronized with the stimulation repetition frequency. The data window was defined as the period between the blanking processes, and the window increment was constrained according to the available maximum processing capability. The processing time delay of the proposed method was less than 33.33 ms, which was enough to meet the real-time constraints. This result suggests that the proposed method satisfies the real-time application requirements for a closed-loop control system.

The proposed method shows a high decoding accuracy in terms of the *R*^2^ values for all of our experiments, where the proprioceptive afferent signals were obtained from anesthetized rabbits during passive limb movements. However, in volitional movements in the un-anesthetized state, muscle spindle firing can be strongly influenced by variations in fusimotor drive and muscle contraction. These influences are absent in the anesthetized animal, but they would be present in practical FES applications. Consequently, the proposed method would likely have a lower decoding performance during active movements resulting from volitional activities or electrical stimulation of motor nerves. In further studies, the decoding performance of the proposed method will be assessed under practical closed-loop FES conditions.

## Methods

### Signal Processing Procedure

**Figure 3:**
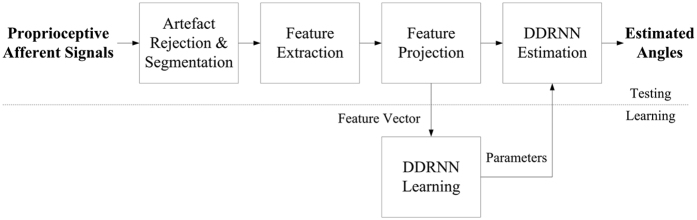
Block diagram of the proposed multiunit activity-based ankle and knee joint angle estimation method based on dorsal root ganglion recordings. In the learning phase, feature vectors were extracted and projected from artefact-rejected and segmented proprioceptive afferent signals. Then, feature vectors were used to learn the DDRNN and determine the parameters. In the testing phase, artefact rejection, segmentation, feature extraction, and projection were applied to a new set of proprioceptive afferent signals. Then, feature vectors were delivered as input to the DDRNN, and ankle and knee joint angles were estimated.

Figure 3 shows a block diagram of the proposed multiunit activity-based angle estimation method based on dorsal root ganglion recordings. Stimulus artefacts were eliminated using a blanking process for each of the 16 channels, and the data window was segmented into a period between the blanking steps. Feature vectors were extracted based on MAVs from each segmented signal, and its dimensionality was reduced via a principle component analysis (PCA). In the learning phase, dimensionally-reduced feature vectors were used for learning the DDRNN to determine the learning parameters, and the DDRNN was constructed to model the relationship between feature vectors and kinematic variables. In the testing phase, the DDRNN was used to estimate ankle and knee joint angles from newly observed proprioceptive afferent signals.

### Dorsal Root Ganglion Recording

Proprioceptive afferent activities are generated by proprioceptors, such as muscle spindles, Golgi tendon organs, joint receptors, and cutaneous receptors that primarily detect changes in muscle displacement, velocity, and force to feedback body movements^23^,^24^. The various muscles act together as agonists and antagonists during limb movement, with contraction and relaxation in opposing muscles being reciprocal, and muscle receptors are simultaneously activated in the muscles. For example, muscle spindles play a major role in detecting position and movement. Muscle stretching leads to increased muscle spindle firing, whereas the return of the muscle from being stretched back to its original length leads to a reduced stretch responses. These responses of muscle spindles are transmitted to the central nervous system as feedback signals that transmit *Kinesthesia* information^23^,^24^. When neural signals were recorded from the dorsal root ganglion, proprioceptive afferent signals were observed during ankle and knee joint movements, and repeatable and consistent neural signal patterns were expected to be observed that correspond to flexion and extension.

All animal experimental procedures were approved by the Institutional Animal Care and Use Committee of the Korea Institute of Science and Technology (Certificate number: AP-2013L1001). The experimental protocol was performed in accordance with the recommendations for the care and use of laboratory animals. Five adult male New Zealand white rabbits, weighing 2–2.3 kg, were used in the experiments. The rabbits were anesthetized via intramuscular injections of a mixture of ketamine (50 mg/kg) and xylazine (10 mg/kg), and anaesthesia was maintained by administering additional doses as needed. The spinal cord and dorsal root ganglion were exposed via laminectomy, and the rabbit was then positioned on a custom-made stereotaxic frame using vertebral clamps. Multichannel microelectrode recordings can provide information about the activity of both individual and ensemble neurons and are therefore considered to be a practical method for representing and correlating information between neuronal responses and behaviours. Neural signals were recorded using a 16-channel microelectrode (A1 × 16–5 mm-50-703-CM16, NeuroNexus Technologies, Ann Arbor, MI, USA), which was inserted perpendicularly into the right L7 ganglion. Each electrode was composed of 16 iridium channels with a 30-μm diameter arranged in a 1 by 16 layout with 50-μm interchannel distances on a 5-mm-long single shank silicon substrate. Reference and ground wires were placed subcutaneously in the back. Neural signals were digitized at 24 kHz and bandpass filtered between 300 and 5000 Hz through a digital data acquisition system (Neuralynx, Tucson, AZ, USA).

**Figure 4:**
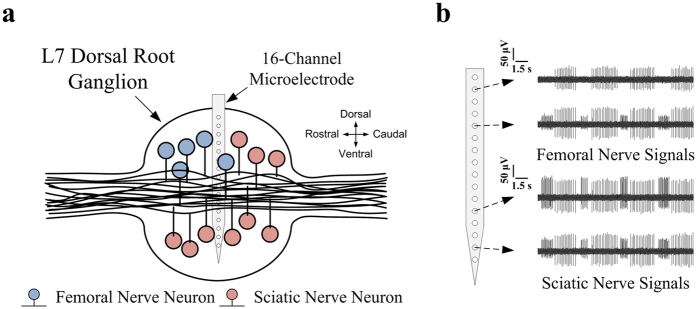
Scheme for proprioceptive afferent signal recording from a dorsal root ganglion using a single-shank microelectrode. (**a**) Simplified illustration for the femoral and sciatic nerve neurons distribution of the L7 dorsal root ganglion in sagittal cross section. The electrode was inserted perpendicularly into the centre of the L7 dorsal root ganglion, where the neurons of the femoral and sciatic nerves are distributed in different regions. (**b**) Each channel was assigned to a different region that corresponded to the areas of the neurons of the femoral and sciatic nerves. The femoral and sciatic nerve signals were simultaneously recorded from the different electrode channels.

Figure 4 shows a scheme for electrode implantation in the L7 dorsal root ganglion of the rabbit. The femoral nerve innervates knee extensors and hip flexors, and the sciatic nerve innervates knee flexors, ankle dorsi-flexors, and ankle plantar-flexors. The proprioceptive sensory fibres of the femoral nerve project to the L5, L6, and L7 dorsal root ganglia, while those of the sciatic nerve project to the L7, S1, and S2 dorsal root ganglia^25^. Femoral and sciatic afferent nerves are both present in the L7 dorsal root ganglion, but there are topographical differences in the distribution of their neurons. Anatomically, femoral nerve neurons are found mainly in the dorsal and rostral regions, and sciatic nerve neurons are found in the medial and ventral regions^26^. When the single-shank microelectrode was perpendicularly inserted into the centre of the L7 dorsal root ganglion, each channel of the electrode was specifically located within different regions of the L7 dorsal root ganglion. As shown in Fig. 4(b), femoral nerve signals were primarily recorded by the upper channels, whereas sciatic nerve signals were commonly recorded by the lower channels. Consequently, the single-shank microelectrode could simultaneously record neural signals from neurons in femoral and sciatic nerves within the L7 dorsal root ganglion.

### Passive Ankle and Knee Joint Movements

**Figure 5:**
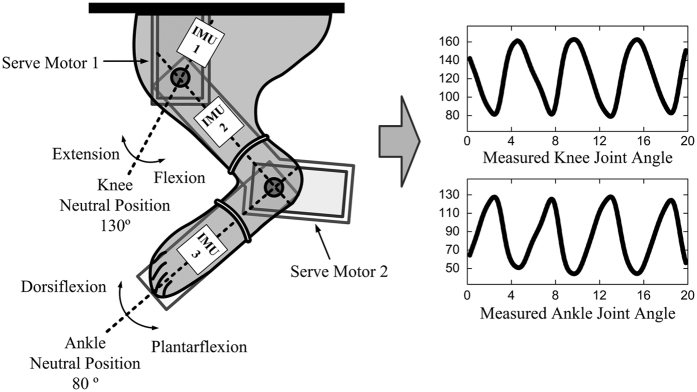
Experimental setup for passive ankle and knee joint movements. The animal was positioned on the custom-made stereotaxic frame. The limb was fastened to the plate with a belt, and the joints were passively moved using the servo motors. The IMU sensors were attached to each segment to measure ankle and knee joint angles.

Figure 5 shows an experimental setup for measuring passive ankle and knee joint movements. A custom-made stereotaxic frame was designed to move the right hind limb, including the ankle and knee joints, in sagittal plane. The ankle and knee joints were passively rotated using servo motors (HS-77BB, Hitec, Korea). The motor shaft was aligned with the ankle and knee joint axis, and the hind limb was fastened with a belt onto the plate. The passive joint motions used here were adopted from the sit-to-stand manoeuvre^27^, where standing is caused by ankle plantarflexion and knee extension, and sitting is caused by ankle dorsiflexion and knee flexion. Repeated sit-to-stand manoeuvres were controlled by a servo controller, which was programmed to generate continuously sinusoidal movement patterns at 0.2 Hz while considering the range of motion (ROM) of the ankle and knee joints. The neutral ankle and knee joint angles were defined as 80 degrees and 130 degrees, respectively, and were based on the limb’s naturally flaccid position on the frame. The ankle and knee joints were moved from 50 to 130 degrees and from 80 to 160 degrees, respectively, considering the maximum ROM of each joint. Joint angles were measured simultaneously with the neural signals using wireless inertial measurement unit (IMU) sensors (EBIMU24G, E2BOX, Seoul, Korea). The IMU sensors were mounted on the right side of the thigh, shank and foot. Measurement data were transmitted to the data acquisition system through a radio frequency receiver at a 60-Hz sampling rate. To synchronize the neural signals with the joint angle data, a transistor-transistor logic pulse output was fed to the digital input of the data acquisition system at the start and end of the recording sessions. Each session was composed of alternating passive flexion and extension periods and continued for 20 s. Joint angle data were collected from five rabbits, and ten sessions were conducted for each rabbit.

### Artefact Rejection and Segmentation

**Figure 6:**
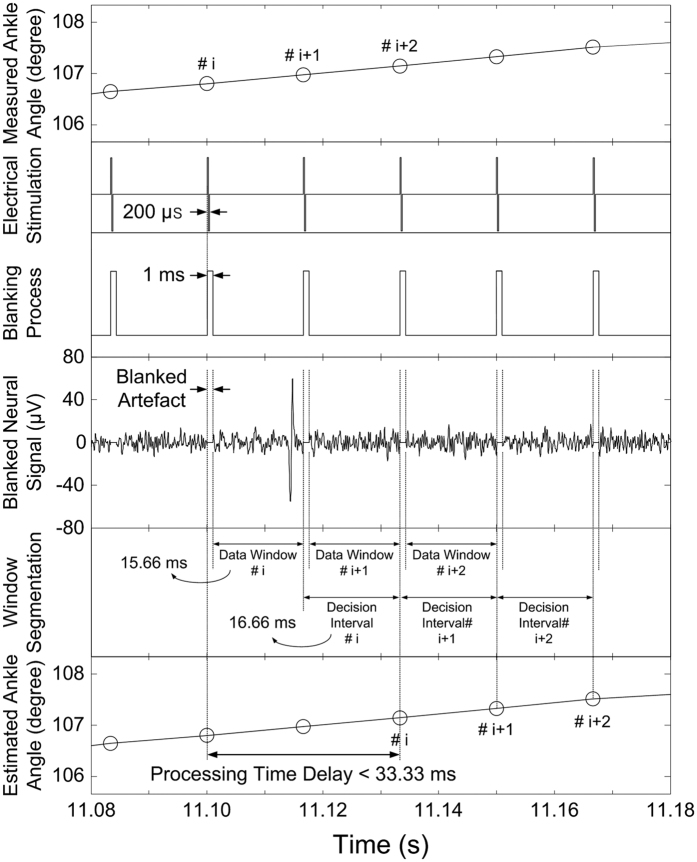
Scheme of the proposed blanking process and data window segmentation. The stimulation repetition frequency was set to be 60 Hz, and a pulse duration of 200 μs was chosen. The blanking process synchronized to the stimulation was applied to eliminate the stimulus artefact. From the blanked neural signals, the data window and decision interval were set to 15.66 ms and 16.66 ms, respectively. As a result, the processing time delay was restricted to be within 33.33 ms.

In FES applications, electrical pulses excite peripheral nerves or muscles, and targeted muscles are contracted as a consequence of electrical stimulation. Concurrently, stimulus artefacts are created by the electrical stimulation and appear in almost all recording channels. These stimulus artefacts interfere with the recording and analysing of neural signals, which may be contaminated by stimulus artefacts such as overlap and distortion. Figure 6 shows a scheme of the blanking process for artefact rejection and data window segmentation. It was assumed that limb movements were generated by electrical stimulation. The stimulation repetition frequency and biphasic current pulse duration were set to 60 Hz and 200 μs, respectively. Stimulus artefacts were rejected using the blanking processes, where the data was discarded for a duration of 1 ms (24 samples) in a manner synchronized with the electrical stimulation^14^. From the result of the blanking process for each channel, the data window was defined as a period of 15.66 ms (376 samples) corresponding to the time between the blanking steps, and feature vectors were extracted from each of these windows. For real-time implementation, all of the angle estimation processes must be completed within the decision interval period. The decision interval was set to 16.66 ms based on a stimulation repetition frequency of 60 Hz. As a result, in the proposed data window segmentation, the processing time delay was restricted to be within 33.33 ms.

### Feature Extraction

**Figure 7:**
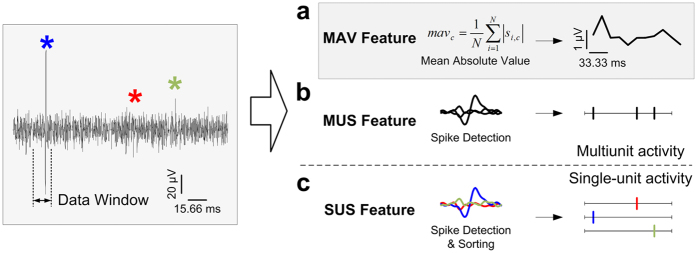
Diagram of the proposed MAV feature extraction method. Decoding performance of the MAV feature was compared with those of two other features, the MUS feature and the SUS feature. (**a**) MAV feature vectors were calculated based on the average of the absolute value of the signal in each data window. (**b**) MUS feature vectors were based on counts of unsorted threshold-crossing events for each channel. (**c**) SUS feature vectors were calculated from the number of spikes of isolated units.

Feature vectors can be categorized based on the number of neurons assumed to be spiking in the recorded neural signals and are classified as either single-unit activity-based feature vectors and multiunit activity-based feature vectors. Generally, a single-unit activity-based feature vector provides good decoding results because it can provide more detailed information about neural signals than a multiunit activity-based feature vector. However, high computational costs and complex algorithms are necessary to determine the activity of individual neurons from signals derived from multiple neurons. Consequently, a fast, simple, and robust extraction method for obtaining limb-state information is required to estimate ankle and knee angles in real time. Hence, we investigated different feature extraction methods to find an optimal solution with a higher decoding performance and less computational effort. Figure 7 shows a diagram of the proposed feature extraction method. Three different features were extracted from the proprioceptive afferent signals. The MAV and multi-unit spike (MUS) feature vectors were constructed from multiunit activity data without spike sorting, whereas single-unit spike (SUS) feature vectors were constructed from single-unit activity data with spike sorting.

First, the MAV was used as a multiunit activity-based feature vector. MAVs have been widely used in bio-signal processing, such as in electromyogram and electroneurogram analyses, which can be easy to implement and simple to use because they are calculated based on raw neural signals in a time series. Additionally, calculating MAVs does not require setting threshold values to detect spikes. MAVs are calculated as the average of the absolute value of the signal in each 15.66-ms bin corresponding to the data window period for each channel, which reflects the activity of the signal. The MAV is defined as


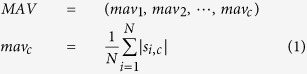


where *mav*_*c*_ is the MAV of the *c***-**th channel in each bin, *N* is the number of samples in each bin and *s*_*i,c*_ is the *i*-th sample in the *c***-**th channel. MAVs were smoothed using a fourth-order Butterworth low-pass filter with a cut-off frequency at 1.67 Hz to convert discrete data into time series of data.

Second, MUSs were constructed from the multiunit activity for each channel. To extract MUSs, each channel was treated as a single putative neuron. The spikes were detected based on threshold crossing events for each channel. The threshold was set to the mean of the baseline noise plus three times the standard deviation of the mean value for each channel. Each spike was composed of 38 sample points corresponding to a spike duration of 1.6 ms, and the refractory period was set to 0.5 ms to prevent counting the same spike twice. The number of threshold crossing events was calculated as the MUS value in each 15.66-ms bin corresponding to the data window period for each channel. The MUS value is defined as


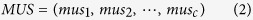


where *mus*_*c*_ is the number of spikes of the *c***-**th channel in each bin. MUS values were smoothed using a fourth-order Butterworth low-pass filter with a cut-off frequency at 1.67 Hz to convert discrete data into time series of data.

Third, a SUS was considered as a single-unit activity-based feature vector, which was defined as the number of spikes from individual neurons. Spikes were detected in the same way as described for MUSs. Spike sorting was performed based on a PCA and *k*-means clustering. A PCA is commonly used for feature extraction and dimensionality reduction for spike sorting. The original spike data were projected onto a new set of coordinates corresponding to the directions of the maximum variance of the data via a linear orthogonal transformation, where a number of correlated variables were transformed into a smaller number of uncorrelated variables. The *k*-means clustering analysis partitions the data set into *k* clusters, where the data points are assigned to a cluster based on the Euclidean distances from the cluster centroids. The first three principal components were used, and the number of clusters was set to vary from 2 to 4. From the spike sorting results, SUS values were calculated for each isolated unit by counting the number of spikes in each 15.66-ms bin corresponding to the data window period. The SUS value is defined as


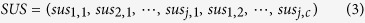


where *sus*_*j,c*_ is the number of spikes of the *j***-**th neuron in the *c***-**th channel for each bin. SUS values were smoothed using a fourth-order Butterworth low-pass filter with a cut-off frequency at 1.67 Hz to convert discrete data into time series of data.

### Feature Projection

Feature projection can reduce the dimensionality of a feature vector by transforming it from the original feature space into an appropriate subspace. Generally, the high dimensionality of a feature vector increases the learning parameters, the processing time, and the risk of overfitting. Therefore, dimensionality reduction is important for improving learning capabilities and decoding accuracy. A PCA was used to reduce the dimensionality of the feature vectors by projecting them into a lower dimensional subspace. The dimensionality of the projected feature vectors was determined by examining their linear dimensionality, which was calculated as the ratio of the sum of the *D* largest eigenvalues to the sum of the total eigenvalues of the covariance matrix. If the ratio was more than 0.97, the linear dimensionality was obtained as *D*^20^. From the results of this rule-based decision, the linear dimensionality was *D* = 3 for all three feature vectors. For example, a dimensionality of 44 was generated for the SUS feature vectors from rabbit A, and this dimensionality was then reduced to 3 via the PCA.

### Neural Decoding of Limb States

To model the relationship between proprioceptive afferents signals and limb states, three different estimators were investigated as candidates for limb-state estimators. The DDRNN was employed as a nonlinear recursive estimator, whereas a multivariate linear regression (MLR), and a Kalman filter were used as linear estimators.

**Figure 8:**
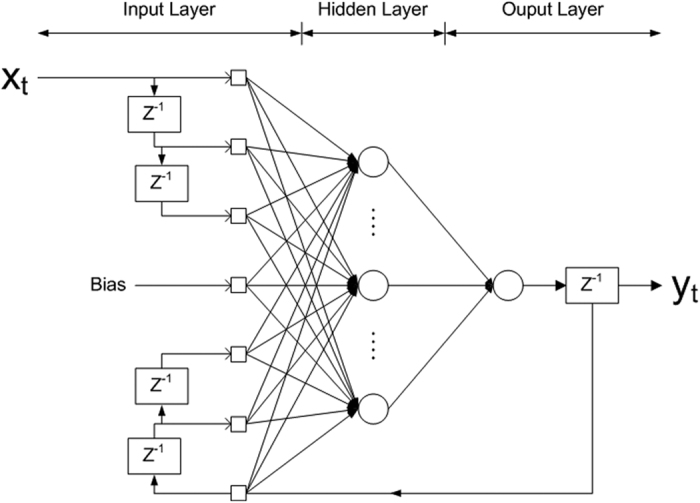
Scheme of the proposed DDRNN structure with a time delay for input and output feedback. The input layer was constructed from the fifteen inputs corresponding to a three-dimensional reduced feature vector with a three-order time delay and a two-dimensional output feedback with a three-order time delay. The hidden layer consisted of twenty neurons, and the output layer consisted of two neurons corresponding to the estimated ankle and knee joint angles.

The DDRNN has proven useful in modelling dynamic systems. Unlike feedforward networks, it involves a recurrent feedback connection from the output to the input, where an internal feedback loop provides the dynamic response of the system, and its network can store information for future reference and learn temporal and spatial patterns^28^. As a result, the network enables the encoding and integration of temporal information, allowing it to model nonlinear dynamic input-output relations. The DDRNN is defined as


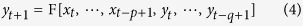


where *x*_*t*_ and *y*_*t*_ are the input and output data at time *t*, respectively, and *p* and *q* are the time-delay order for the input and output feedback, respectively. F is a nonlinear mapping function. The future output data, *y*_*t*+1_, is predicted from the present and past input data, 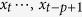, and the present and past output data, 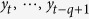. The DDRNN was characterized by an input layer, a hidden layer, and an output layer. For DDRNN learning, two time-delay orders need to be set; one is the time delay order for the input, and the other is the time delay order for the output feedback. Generally, a high delay order for the input increases input dimensionality and computational complexity. Additionally, a high delay order for the output feedback frequently causes oscillations, divergence, or instability in the networks and leads to unsatisfactory performance^29^. Therefore, optimal values for the time delay orders for both the input and output feedback should be chosen to reduce processing times and improve decoding performance. Figure 8 shows the structure of the proposed DDRNN estimator. The network structure was optimized via a trial-and-error procedure, and the connecting weights and biases were adjusted using an error back-propagation algorithm. The values for *p* and *q* were set to the three-order time delay based on the investigation of the different delay orders. As a result, the input layer consisted of fifteen neurons corresponding to a three-dimensional reduced-feature vector with a three-order time delay and a two-dimensional output feedback with three-order time delay. The hidden layer consisted of twenty neurons, with the nodes of the input layer fully connected to the nodes of the hidden layer. The output layer was set to two neurons for the estimation of ankle and knee joint angles.

A MLR estimates state variables based on a weighted summation of two or more observation variables. A MLR model is defined as^9^,^10^


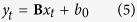


where *y*_*t*_ and *x*_*t*_ are the state and observation variables at time *t*, respectively. *b*_0_ is the intercept in the regression model, and **B** is the coefficient matrix. Ankle and knee joint angles correspond to state *y*_*t*_, whereas the three-dimensional reduced-feature vector corresponds to the observation *x*_*t*_. The values for *b*_0_ and **B** were determined from a least-squares fit to the learning data.

A Kalman filter recursively infers state variables from the observation variables. The state is assumed to be a linear function of the state at the previous time plus Gaussian noise, and the observation is assumed a linear function of the state plus Gaussian noise. The state and observation models are defined as^30^


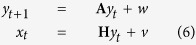


where *y*_*t*_ and *x*_*t*_ are the state and observation variables at time *t*, respectively, and *w* and *v* are the process and measurement noise, respectively. **A** and **H** are the coefficient matrices of the state and observation models, respectively. The *w* and *v* values were assumed to be zero mean and normally distribution. **W** and **V** are the covariance matrices of the process and measurement noise, respectively. The ankle and knee joint angles correspond to the state *y*_*t*_, whereas the three-dimensional reduced-feature vector corresponds to the observation *x*_*t*_. The **A** and **H** values were estimated from the learning data using a least-squares estimation. Estimate of *y*_*t*_, 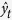, was obtained using the following equation:


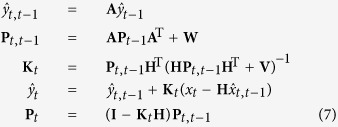


where 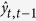 is the priori estimate from the previous time, *t* − 1, and **P**_*t,t*−1_ is the priori error covariance matrix. 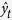 is the posteriori estimate updated using the 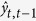 and *x*_*t*_ values. **P**_*t*_ is the posteriori error covariance matrix and **K**_*t*_ is the Kalman gain matrix.

### Performance Evaluation

To evaluate the decoding performance of the proposed method for estimating ankle and knee joint angles, proprioceptive afferent signals were recorded from the L7 dorsal root ganglion using a 16-channel microelectrode during the passive movement of the ankle and knee joints. Then, the blanked proprioceptive afferent signals were segmented to a 15.66-ms data window. For each window, the feature vectors were extracted based on the MAV, and their dimensionality was reduced to 3 via a PCA. These dimensionally reduced feature vectors were applied to the DDRNN, which was implemented with a three-order time delay for the input and output feedback.

Decoding performance was determined based on the test data from the five rabbits. For each rabbit, the decoding accuracy was evaluated using a 10-fold cross-validation. Of the ten sessions, one session was randomly selected for generating the test data, and the remaining nine sessions were used to collect learning data. This procedure was repeated ten times (ten-fold). The decoding accuracy was quantified based on the coefficient of determination (*R*^2^), which is the percent the variation between the measured and estimated values. Total decoding accuracy was calculated as the mean of the accuracy in the five rabbits. A two-way analysis of variance (ANOVA) was used to assess the statistical significance of the differences and to remove the effect of difference among the rabbits. A confidence level of 95% (*p* < 0.05) was considered to indicate a significant difference.

### Signals Properties of the Recorded Proprioceptive Afferent Signals

We assumed that when proprioceptive afferent signals were recorded from the L7 dorsal root ganglion using a single-shank microelectrode, the femoral and sciatic nerve signals were being detected by different channels during the ankle and knee joint movements at the same time. To confirm this assumption, neural signals were monitored during ankle or knee joint movements alone.

### Comparison of Different Feature Extraction Methods

To select an optimal feature vector for estimating ankle and knee joint angles, the decoding accuracy was investigated for different feature extraction methods: MAV, MUS, and SUS. Three-dimensional reduced-feature vectors were used as input for the DDRNN, which was optimized using a three-order time delay for the input and output feedback.

### Comparison of Different Delay Orders

To determine optimal delay orders, the decoding accuracy was investigated for different delay orders, where the delay orders of the input and the output feedback were assumed to be equal. The three-dimensional MAV feature was used as the input vector for the DDRNN, and the decoding accuracy was compared among the one-, three-, five-, and seven-delay orders.

### Comparison of Different Decoding Methods

The effect of different decoding methods on decoding accuracy was investigated. The decoding accuracy of the proposed DDRNN was compared to those of other estimators: the MLR and Kalman filter estimators. For the DDRNN, the three-dimensional MAV feature with a three-order time delay and a two-dimensional output feedback with a three-order time delay was used as the input. For the MLR, a three-dimensional MAV feature with a three-order time delay was used as the observation variable, and two-dimensional ankle and knee joint angles were used as the state variables so that the intercept was set to 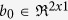 and the dimension of the matrix was set to 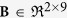. For the Kalman filter, a three-dimensional MAV feature with a three-order time delay was used as the observation variable, and two-dimensional ankle and knee joint angles with a three-order time delay were used as the state variables. Therefore, the dimensions of the matrices were set to 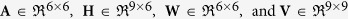.

### Decoding Performance of the Proposed Method

To evaluate the functional performance for real-time implementation, the processing time of the proposed method was investigated for each process step, feature extraction, feature projection, and neural decoding for the test data. The experiment was conducted on an Intel^®^ Core™ i-7 4710HQ 2.5-GHz PC with 8-GB RAM, and processing time was monitored.

## Additional Information

**How to cite this article**: Han, S. *et al*. Multiunit Activity-Based Real-Time Limb-State Estimation from Dorsal Root Ganglion Recordings. *Sci. Rep.* **7**, 44197; doi: 10.1038/srep44197 (2017).

**Publisher's note:** Springer Nature remains neutral with regard to jurisdictional claims in published maps and institutional affiliations.

## Figures and Tables

**Table 1:** Spike sorting results for each rabbit.

Rabbit	A	B	C	D	E
Number of recording channels	14	14	9	9	10
Total number of isolated units	44	43	39	37	36

**Table 2:** Decoding accuracy with respect to ankle joint movement for different feature extraction methods.

Rabbit	MAV	MUS	SUS
A	0.99923	0.99904	0.99990
B	0.99930	0.99868	0.99559
C	0.99968	0.99996	0.99996
D	0.99954	0.99929	0.99988
E	0.99995	0.99996	0.99974
Mean ± SD	0.99954 ± 0.00026	0.99939 ± 0.00051	0.99902 ± 0.00171

*R*^2^ value between actual and estimated angles is given as decoding accuracy.

**Table 3:** Decoding accuracy with respect to knee joint movement for different feature extraction methods.

Rabbit	MAV	MUS	SUS
A	0.99957	0.99976	0.99993
B	0.99741	0.97678	0.99523
C	0.99985	0.99982	0.99999
D	0.99821	0.99796	0.99983
E	0.99996	0.99993	0.99994
Mean ± SD	0.99900 ± 0.00101	0.99485 ± 0.00907	0.99898 ± 0.00188

*R*^2^ value between actual and estimated angles is given as decoding accuracy.

**Table 4:** Decoding accuracy with respect to ankle joint movement for different delay orders.

Rabbit	1-order (16.66 ms)	3-order (50 ms)	5-order (83.33 ms)	7-order (116.66 ms)
A	0.98284	0.99923	0.99714	0.99975
B	0.69136	0.99930	0.97883	0.99384
C	0.95486	0.99968	0.99990	0.99956
D	0.98316	0.99954	0.99875	0.99562
E	0.99913	0.99996	0.99993	0.99986
Mean ± SD	0.92227 ± 0.11633	0.99954 ± 0.00026	0.99491 ± 0.00810	0.99773 ± 0.00251

*R*^2^ value between actual and estimated angles is given as decoding accuracy.

**Table 5:** Decoding accuracy with respect to knee joint movement for different delay orders.

Rabbit	1-order (16.66 ms)	3-order (50 ms)	5-order (83.33 ms)	7-order (116.66 ms)
A	0.96713	0.99957	0.99912	0.99921
B	0.89786	0.99741	0.99105	0.99878
C	0.96807	0.99985	0.99998	0.99973
D	0.95663	0.99821	0.99859	0.99541
E	0.99790	0.99996	0.99982	0.99992
Mean ± SD	0.95752 ± 0.03285	0.99900 ± 0.00101	0.99771 ± 0.00337	0.99861 ± 0.00165

*R*^2^ value between actual and estimated angles is given as decoding accuracy.

**Table 6:** Decoding accuracy with respect to ankle joint movement for different decoding methods.

Rabbit	DDRNN	MLR	Kalman filter
A	0.99923	0.24700	0.30779
B	0.99930	0.55617	0.63063
C	0.99968	0.59771	0.76142
D	0.99954	0.77721	0.83846
E	0.99996	0.48840	0.67892
Mean ± SD	0.99954 ± 0.00026	0.53204 ± 0.30592	0.60560 ± 0.29205

*R*^2^ value between actual and estimated angles is given as decoding accuracy.

**Table 7:** Decoding accuracy with respect to knee joint movement for different decoding methods.

Rabbit	DDRNN	MLR	Kalman filter
A	0.99957	0.23619	0.30608
B	0.99741	0.56551	0.63814
C	0.99985	0.64376	0.75979
D	0.99821	0.78092	0.83953
E	0.99996	0.49678	0.66936
Mean ± SD	0.99900 ± 0.00101	0.54330 ± 0.30717	0.60429 ± 0.29093

*R*^2^ value between actual and estimated angles is given as decoding accuracy.

**Table 8:** Processing time for the proposed method.

Processes	Processing Time (ms)
MAV	4.49
PCA	4.50
DDRNN	5.55
Total	14.54
